# Respiratory syncytial virus hospitalization outcomes and costs of full-term and preterm infants

**DOI:** 10.1038/jp.2016.113

**Published:** 2016-08-04

**Authors:** K K McLaurin, A M Farr, S W Wade, D R Diakun, D L Stewart

**Affiliations:** 1 Health Economics and Outcomes Research, Medical Affairs, AstraZeneca, Gaithersburg, MD, USA; 2 Life Sciences, Truven Health Analytics an IBM Company, Cambridge, MA, USA; 3 Wade Outcomes Research and Consulting, Salt Lake City, UT, USA; 4 Life Sciences, Truven Health Analytics, an IBM Company, Bethesda, MD, USA; 5 Department of Pediatrics, University of Louisville School of Medicine, University of Louisville Hospital, Kosair Children's Hospital, Louisville, KY, USA

## Abstract

**Objective:**

Infection with respiratory syncytial virus (RSV), which causes lower respiratory tract infections, is the leading cause of hospitalization among children <1 year old in the United States. Risk factors for RSV hospitalization include premature birth and younger chronologic age, along with several comorbid conditions. However, in terms of RSV hospitalization costs, premature infants are rarely studied separately from full-term infants. The objective of this study is to describe the cost and severity of RSV hospitalizations among preterm and full-term infants without chronic lung disease or other high-risk conditions.

**Study Design:**

This analysis used Truven Health Market Scan Multi-State Medicaid and Commercial Claims and Encounters databases, which contain a combined 4 million births from 2003 to 2013. Infants with comorbid conditions associated with increased risk for RSV infection were excluded. Infants were classified as preterm (<29, 29−30, 31−32, 33−34 and 35−36 weeks' gestational age (wGA)) or full term based on diagnostic coding. Health-care claims during the first year of life were evaluated for RSV hospitalizations, defined as inpatient claims with a diagnosis code for RSV in any position. Costs of RSV hospitalizations were captured and reported in 2014 USD. Inpatient claims for RSV hospitalizations were evaluated for the presence of codes indicating admission to the intensive care unit (ICU), use of mechanical ventilation (MV) and length of stay. These three measures were used to describe hospital severity. Chronologic age at the time of RSV hospitalization was also captured. Data were summarized and no statistical comparisons were conducted.

**Results:**

There were 1 683 188 infants insured through Medicaid and 1 663 832 infants insured through commercial plans born from 1 July 2003 to 30 June 2013. Of those, 10.8 and 8.8% in each database, respectively, were born prematurely. There were 29 967 Medicaid-insured infants and 16 310 commercially insured infants with an RSV hospitalization during their first year of life. Mean first-year RSV hospitalization costs were higher for preterm infants, ranging from $8324 and $10 570 for full-term infants to $15 839 and $19 931 for preterm infants 33–34 wGA, and to $39 354 and $40 813 for preterm infants <29 wGA, among Medicaid-insured and commercially insured infants, respectively. RSV hospitalizations also tended to be more severe among preterm infants, with longer lengths of stay, a higher proportion of infants admitted to the intensive care unit (ICU) and increased use of MV compared with full-term infants. Mean costs of RSV hospitalizations with a PICU admission ranged from approximately $35 000 to $89 000. In both Medicaid and commercial groups, costs were greater for infants hospitalized at <90 days of age compared with older infants.

**Conclusions:**

Infants who were born prematurely and those hospitalized at <90 days of age have more costly and more severe RSV hospitalizations during the first year of life. These findings demonstrate important differences in the costs and severity of first-year RSV hospitalizations of premature and full-term infants. These differences are likely to be obscured in combined analysis, in which full-term infants predominate. Clinical guidelines and health-care policies relating to RSV would benefit from the availability of data obtained from separate analyses of these two infant subgroups.

## Introduction

Respiratory syncytial virus (RSV) is a common virus that often causes lower respiratory tract infections (LRTIs). LRTIs may cause respiratory distress including tachypnea, wheezing, retractions and cyanosis, as well as other serious complications, and result in more than 100 000 hospitalizations of children <1 year of age in the United States every year.^1^ Among infants, RSV is the leading cause of hospitalization, and infants born prematurely are at increased risk for severe RSV disease and mortality compared with full-term infants.^2, 3, 4^ Other high-risk groups include infants with bronchopulmonary dysplasia/chronic lung disease of prematurity (BPD/CLD), hemodynamically significant congenital heart disease (HS-CHD) and other congenital abnormalities.^5^

RSV immunoprophylaxis with palivizumab has been approved by the US Food and Drug Administration since 1998 for the prevention of severe RSV disease. It is indicated for use in preterm infants born at ⩽35 weeks' gestational age (wGA) and ⩽6 months of age at the start of the RSV season, children ⩽24 months of age who have BPD/CLD and children ⩽24 months of age with HS-CHD.^6^

In recent years, the American Academy of Pediatrics' guidance for RSV prophylaxis has changed. The 2014 guidance does not recommend palivizumab for either 29–35 wGA infants without BPD/CLD or HS-CHD, or children with HS-CHD who are in their second RSV season. This significant change was due in part to ongoing concern about the cost-effectiveness of RSV immunoprophylaxis.^7^ However, some of the data used in prior analyses reflect the experience of all infants and are not specific to high-risk infants. For example, a recent study that summarized trends in bronchiolitis hospitalizations reported an average cost of $8530.^8^ Other researchers have assumed that this figure was applicable to all palivizumab-eligible infant populations,^9^ when in fact this is the average cost of hospitalizations for infants <2 years of age with bronchiolitis, and is neither specific to RSV nor to high-risk infants. Summary statistics such as this are unlikely to accurately reflect the experience of high-risk infants, who represent a small proportion of the total infant population.

Previous studies have documented that RSV hospitalizations are associated with greater resource utilization and costs during the first year of life among preterm infants compared with full-term infants.^10, 11, 12^ However, only one of these studies examined RSV hospitalization-specific outcomes, and none of the studies researched either preterm infant subgroups more specific than <33 wGA and 33–36 wGA or both publically and commercially insured populations. Therefore, the objective of this study was to provide contemporary and detailed information about the cost and severity of RSV hospitalizations among preterm and full-term Medicaid and commercially insured infants without CLD or other high-risk conditions.

## Methods

### Data sources

This analysis used data from Truven Health MarketScan Multi-State Medicaid and Commercial Claims and Encounters. These data are composed of inpatient and outpatient medical and outpatient pharmacy claims and encounters. Information on patient enrollment and demographics is also included. Claims and patient information are linked using a unique enrollee identifier. Patients are covered under a variety of fee-for-service and managed care plans. The Medicaid database comprised multiple, geographically dispersed states. From 2003 to 2013, the two databases included more than 4 million births. All claims are fully adjudicated and paid. Data are fully compliant with the Health Insurance Portability and Accountability Act of 1996. Institutional Review Board approval was not required because this study did not involve data collection and the data are de-identified.

### Patient sample

The study population consisted of Medicaid-insured and commercially insured infants who were born between 1 July 2003 and 30 June 2013. Infants were identified by inpatient claims for their birth hospitalization using diagnosis-related group (DRG) codes and *International Classification of Diseases, Ninth Revision, Clinical Modification* (*ICD-9-CM*) diagnosis codes. Only infants who were discharged from their birth hospitalization alive were included in this analysis. Infants at high risk for RSV for reasons other than premature birth (BPD/CLD, HS-CHD, cystic fibrosis, trisomy 21, immunodeficiencies or organ transplants) were excluded. The remaining infants were characterized as preterm or full term based on *ICD-9-CM* diagnosis codes and DRG codes. An infant's claims from birth through the end of the first year of life were initially evaluated for the presence of an *ICD-9-CM* diagnosis code indicating gestational age, and then evaluated for the presence of a DRG code indicating full-term status. Data for infants with no evidence of either an *ICD-9-CM* diagnosis for gestational age or a DRG code for full-term birth were excluded because these infants could not be classified as either premature or full term.

### Outcome variables

Infants were followed from birth to the first of the following events: end of continuous enrollment in their insurance, end of study period or end of the first year of life. During follow-up, RSV hospitalizations were identified by *ICD-9-CM* diagnosis codes 466.11, 480.1 and 079.6 in any position on an inpatient claim with a date of service occurring any time >1 day after the birth discharge date. Hospitalization costs were calculated based on the paid amount on the claim, which included both insurer and patient out-of-pocket costs, and were adjusted to 2014 USD using the medical component of the Consumer Price Index. Hospitalizations with $0 cost were excluded from summary cost statistics. The first RSV hospitalization for each infant was analyzed to approximate hospitalization severity using three indicators: length of stay, admission to an intensive care unit (ICU) and use of mechanical ventilation (MV). ICU and MV use were identified using relevant procedure and revenue codes. The infant's chronologic age at the time of first RSV hospitalization was measured using birth date and admission date. The proportion of infants with at least one palivizumab dose was determined by evaluating outpatient medical and outpatient pharmacy claims for procedure codes and drug codes for palivizumab.

### Analysis

This study was descriptive in nature, and statistical comparisons were not conducted. Costs of RSV hospitalizations during the first year of life and RSV hospitalization characteristics were summarized using means, standard deviations, medians and interquartile ranges for continuous variables, and counts and proportions for categorical variables. Results were stratified by preterm and full-term status and by chronologic age at the time of RSV hospitalization. Data for infants insured through commercial plans and infants insured through Medicaid plans were analyzed separately. Analyses were conducted with SAS 9.3 (Cary, NC, USA).

## Results

**Figure 1:**
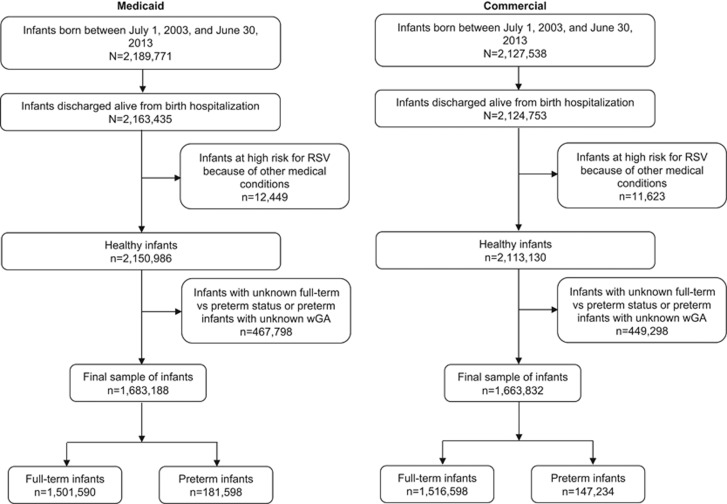
Sample selection flow chart. RSV, respiratory syncytial virus; wGA, weeks' gestational age.

There were 2 163 435 and 2 124 753 infants identified in the Medicaid and commercial insurance databases, respectively. Sample attrition is presented in Figure 1. Approximately 12 000 infants in each database were excluded because they were determined to be at high risk for RSV for reasons other than gestational age at birth. In the Medicaid sample, 2565 (0.12%) were classified as BPD/CLD and 4818 were classified as HS-CHD (0.22%). In the commercial sample, 2292 (0.11%) infants were classified as BPD/CLD and 3775 (0.18%) were classified as HS-CHD. Overall, ~1.7 million infants from each database could be classified as either preterm or full term based on available gestational age information or reported status at birth. Of these infants, 10.8% (*n*=181 598) of Medicaid-insured infants were classified as preterm and 89.2% (*n*=1 501 590) were classified as full term. In the commercial sample, 8.8% (*n*=147 234) of infants were classified as preterm and 91.2% (*n*=1 516 598) were classified as full term. There were 29 967 Medicaid-insured infants and 16 310 commercially insured infants with an RSV hospitalization during follow-up (Table 1). The proportion of infants with an RSV hospitalization was higher among preterm infants compared with full-term infants and also higher among Medicaid-insured infants compared with commercially insured infants. A small proportion of infants hospitalized for RSV (5.1% in the Medicaid population and 4.5% in the commercially insured population) had more than one RSV hospitalization during follow-up. Therefore, the total number of RSV hospitalizations was 31 602 in the Medicaid group and 17 101 in the commercially insured group. The proportion of infants with at least one palivizumab dose was similar in the two populations; between 40 and 56% of infants born at <33 wGA had at least one dose, compared with 19% of 33–34 wGA infants, 3% of 35–36 wGA infants and <1% of full-term infants.

The majority of first RSV hospitalizations occurred between November and March (88.2% in the Medicaid population and 89.5% in the commercially insured population). Average chronologic age at first RSV hospitalization decreased with increasing gestational age. Average chronologic age at first RSV hospitalization among infants born at <29 wGA was 182.8 days (s.d. 83.9) and 198.5 days (s.d. 72.8) among Medicaid-insured and commercially insured infants, respectively. Among full-term infants, average chronologic age at first RSV hospitalization was 116.3 days (s.d. 92.4) among Medicaid-insured infants and 110.7 days (s.d. 89.3) among commercially insured infants. A diagnosis or DRG code indicating the prematurity status of the hospitalized infant was rarely reported on claims for RSV hospitalizations. Of the total 48 703 RSV hospitalizations identified in the Medicaid and commercially insured populations, 1.3% had a diagnosis code indicating wGA ⩽36 and 3.3% had a diagnosis code or DRG indicating that the infant was full term.

**Figure 2:**
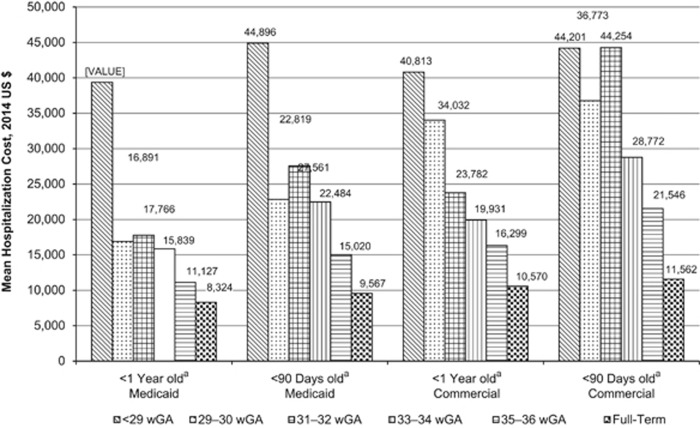
Mean first-year costs of hospitalizations for RSV by gestational age and by age at first hospitalization for RSV. RSV, respiratory syncytial virus; wGA, weeks' gestational age. ^a^Age at the time of first hospitalization for RSV.

**Figure 3:**
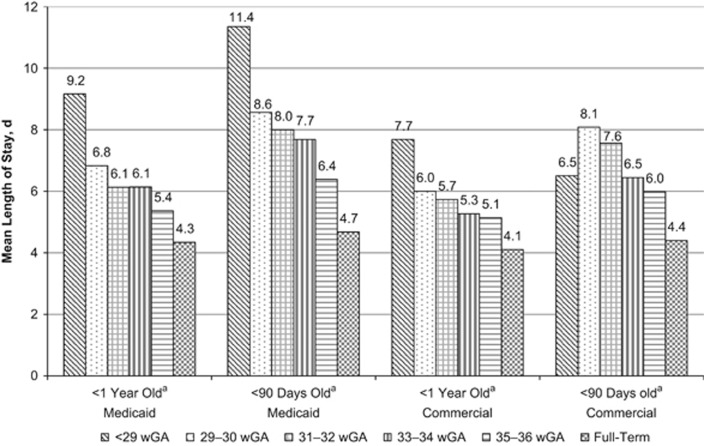
Mean length of stay of hospitalizations for RSV by gestational age and by age at first hospitalization for RSV. RSV, respiratory syncytial virus; wGA, weeks' gestational age. ^a^Age at the time of first hospitalization for RSV.

**Figure 4:**
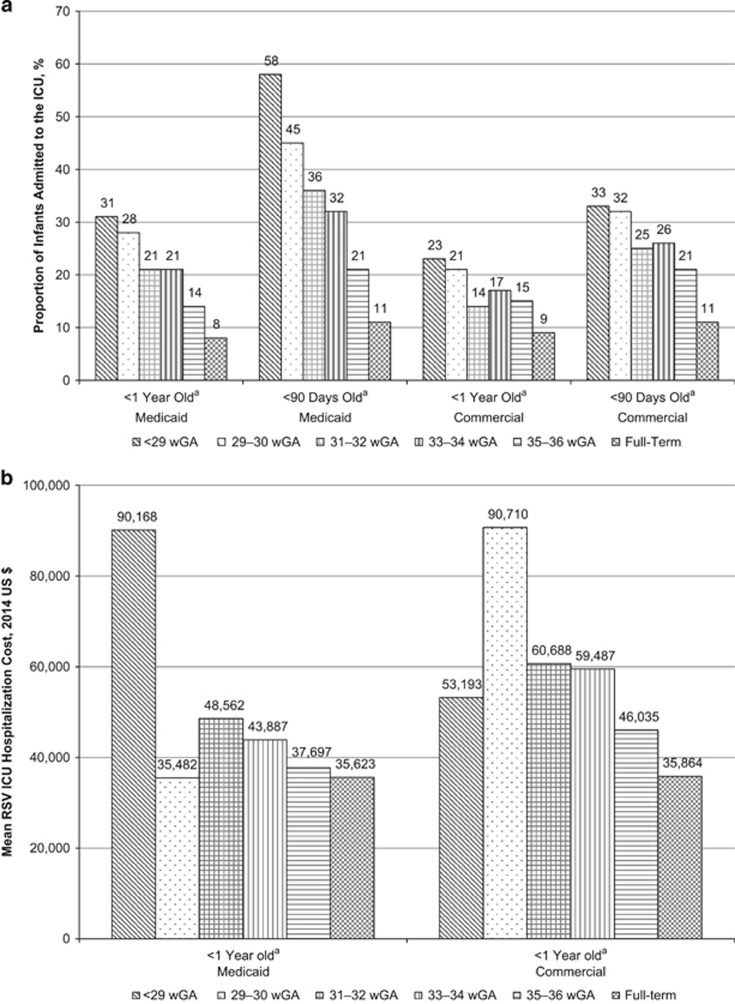
(**a**) Proportion of infants admitted to the ICU during hospitalization for RSV by gestational age and by age at first hospitalization for RSV and (**b**) mean first-year costs of hospitalizations for RSV by gestational age and by age at first hospitalization for RSV during which infant was admitted to the ICU. ICU, intensive care unit; RSV, respiratory syncytial virus; wGA, weeks' gestational age. ^a^Age at the time of first hospitalization for RSV.

**Figure 5:**
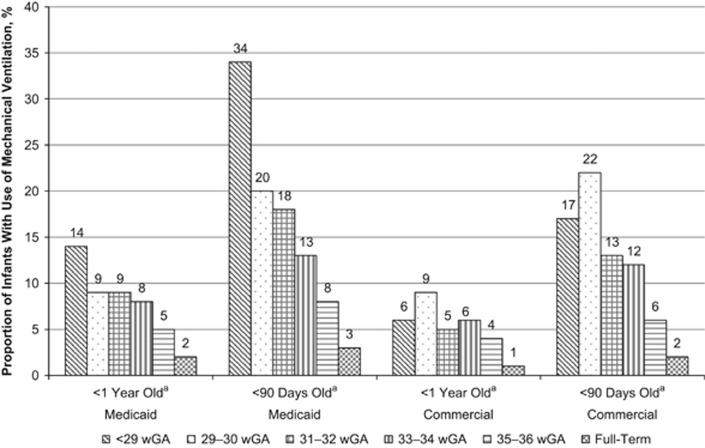
Proportion of infants with use of mechanical ventilation during hospitalization for RSV by gestational age and by age at first hospitalization for RSV. RSV, respiratory syncytial virus; wGA, weeks' gestational age. ^a^Age at the time of first hospitalization for RSV.

Among infants with at least one RSV hospitalization, the mean first-year RSV hospitalization costs increased with decreasing wGA among both Medicaid-insured and commercially insured infants (Figure 2 and Supplementary Table 1). The mean costs of RSV hospitalizations ranged from $8324 among full-term infants to $39 354 among preterm infants <29 wGA in the Medicaid sample. In the commercial sample, mean first-year costs ranged from $10 570 to $40 813. Similarly, RSV hospitalizations were more severe among preterm infants compared with full-term infants; the average length of stay, proportion of infants admitted to the intensive care unit (ICU) and proportion of infants with use of MV increased with decreasing wGA (Figures 3, 4, 5 and Supplementary Table 1). Average length of stay for the first RSV hospitalization was almost 5 days longer for Medicaid infants <29 wGA compared with full-term infants (9.2 vs 4.3 days) and nearly 4 days longer for commercially insured infants <29 wGA compared with full-term infants (7.7 vs 4.1 days).

Nearly one-fifth (19.7%) of preterm infants insured through Medicaid were admitted to the ICU during the first RSV admission compared with 8.0% of full-term infants. Among infants with commercial insurance, 16.3% of preterm infants were admitted to the ICU compared with 8.5% of full-term infants. Rates of ICU admission were highest among infants <90 days of age at the time of their first RSV hospitalization, with rates ranging from 21 to 58% among preterm infants (Figure 4a). The mean cost of RSV hospitalizations was higher among infants admitted to the ICU, with costs ranging from $35 623 and $35 864 among full-term infants in Medicaid and commercial plans, respectively, to $90 168 among Medicaid-insured infants <29 wGA and $90,710 among commercially insured infants 29–30 wGA (Figure 4b). Similarly, evidence of MV increased with decreasing gestational age, and rates of use were often twice as high for infants <90 days old compared with all infants <1 year (Figure 5). The mean cost of RSV hospitalizations among infants who had evidence of MV in addition to a ICU admission was approximately 1.5–2.5 times greater than the mean cost of RSV hospitalizations among those infants admitted to the ICU overall (Supplementary Table 2). There were 20 Medicaid-insured infants who died during the hospitalization stay: 6 (1.0%) <29 wGA, 3 (0.6%) 29–30 wGA, 2 (0.15%) 33–34 wGA, 1 (0.04%) 35–36 wGA and 8 (0.03%) full-term infants. Five commercially insured infants died during the hospitalization stay: 1 (0.6%) <29 wGA and 4 (0.03%) full-term infants.

## Discussion

This retrospective claim-based analysis of Medicaid-insured and commercially insured preterm and full-terms infants found that in the first year of life RSV hospitalizations of preterm infants (<37 wGA) were more costly and more severe than RSV hospitalizations of full-term infants. These differences remained similar for infants who were <90 days of age at the time of their first RSV hospitalization. This study is the first to provide a side-by-side assessment of the costs of hospitalization of full-term vs preterm infants in large populations of Medicaid-insured and commercially insured infants. Infants with other conditions placing them at high risk for RSV were excluded to minimize confounding of inpatient utilization and associated costs.

The risk of RSV hospitalization has previously been shown to increase as gestational age decreases;^1, 2^ this analysis adds to the literature by demonstrating that RSV hospitalization severity also increases as gestational age decreases. Additionally, this analysis includes both preterm and full-term infants in the patient population but analyzes them separately. The severity or intensity of the inpatient stay was reflected by the need for admission to the ICU and/or MV, in addition to length of stay. Although the number of deaths reported was small, the mortality rates among Medicaid-insured infants also increased with decreasing gestational age. The findings in this study are supported by clinical research showing that preterm infants are at an increased risk for severe RSV infection due to small lung volumes, reduced lung surface area, smaller airways and an increased air space wall thickness.^13, 14^ In addition, the immune system in preterm infants is immature, resulting in low antibody titers from maternal transfer and reduced immunity with reduced viral clearance.^15, 16^

Similar trends in RSV hospitalization severity by gestational age were noted in both Medicaid-insured and commercially insured infant samples, with slightly more severe hospitalizations among Medicaid infants. This is consistent with a previous study of LRTIs among children, which found a higher rate of serious hospitalizations, defined as those with a PICU admission or MV, among Medicaid enrollees compared with children with commercial insurance.^17^ The higher rates of RSV hospitalization found in this analysis, along with the more severe characteristics of these hospitalizations, may be due in part to inadequate or delayed care for RSV infection among infants with Medicaid insurance. This may lead to more serious health outcomes; however, these factors cannot be directly assessed in administrative claims data.

Most RSV-infected children admitted to the PICU are not in any recognized high-risk categories,^18^ although several studies have attempted to identify risk factors for severe RSV infection leading to PICU admission. Certain clinical findings are commonly associated with PICU admissions, including retractions, grunting, cyanosis and lung consolidation. Additionally, prolonged length of stay has been associated in other analyses with underlying chronic illness, lung consolidation and need for and duration of oxygen therapy or MV, in addition to PICU admission.^3, 18, 19, 20, 21^

Irrespective of gestational age, RSV hospitalizations that occurred among infants <90 days old were more severe, that is, requiring more intensive and longer treatment, than those occurring among older infants. This finding has been reported by another analysis of RSV hospitalizations among children <5 years old, which estimated that RSV hospitalization rate was highest among infants <3 months old and decreased as children aged.^1^ Infants of young chronologic age may be at higher risk of RSV hospitalization because of a combination of factors: they have smaller airways, lack an experienced immune system and may come into contact with other small children.^22^

More severe RSV hospitalizations among preterm infants translate into more costly RSV hospitalizations for infants during the first year of life. This analysis found that the cost of RSV hospitalizations among preterm infants was 1.3 and 1.5 times greater, respectively, for Medicaid-insured and commercially insured infants born at 35–36 wGA compared with full-term infants. This cost differential increased to 4.7 and 3.9 times greater, respectively, for Medicaid and commercially insured infants born at <29 wGA compared with full-term infants. Average costs for RSV hospitalizations when the infant was admitted to the ICU were almost $36 000 for full-term infants and were substantially higher for preterm infants, reaching a maximum average of $90 000 for infants born at <29 wGA in the Medicaid sample and infants born at 29–30 wGA in the commercial insurance sample. Mean costs tended to be higher still for those infants who were admitted to the PICU and treated with MV. Average costs for Medicaid-insured infants were generally lower than those for commercially insured infants, despite the fact that hospitalizations among Medicaid infants tended to be more severe by the measures used in this analysis. This may be explained by Medicaid reimbursement rates, which have been shown to be lower than reimbursements for other types of insurance.^23^ Future research is needed to explain the differences in severity between Medicaid-insured and commercially insured infants. Possible considerations for future studies include exploring prenatal and postnatal factors that may differ between the two populations, including maternal smoking habits, nutritional factors, inadequate prenatal and perinatal care, crowded living spaces and exposure to the RSV. Unfortunately, these characteristics cannot be captured based on administrative claims data alone; therefore, additional data from other sources would be needed to test these hypotheses.

The cost estimates reported in this analysis are dramatically different from cost estimates often cited in RSV studies in which data for preterm and full-term infants and other high-risk infants have been analyzed as a single aggregate group. A study using the Healthcare Cost and Utilization Project Kids' Inpatient Database reported that in 2009, the mean hospital charge per case of bronchiolitis among patients <2 years of age was $8543 in 2009 USD.^8^ The authors report the mean among children with 'no high-risk condition,' which incorporated children born prematurely and those with other comorbid conditions ($8099), but they do not report estimates for premature infants or those who have other significant risk factors for RSV.^7^ Although an approximation of $8000 has been used as an estimate for the cost of an RSV hospitalization in prophylaxis cost-effectiveness calculations,^9^ this figure is not specific to hospitalizations for RSV, and its use for that purpose is therefore inaccurate. The estimated costs used in such calculations should be specific to gestational age and health condition. Additionally, it is important to evaluate more than just the RSV hospitalization data to determine gestational age. As this analysis demonstrates, gestational age is not commonly coded on RSV hospitalization claims.

### Limitations

This analysis has several limitations. The infants included may have received palivizumab prophylaxis during their first year of life, which would have influenced the outcomes observed for preterm infants, who are more likely to receive prophylaxis. Palivizumab administered during birth hospitalization could not be captured; therefore, prophylaxis rates may be underestimated, particularly for preterm infants, who are likely to receive prophylaxis before discharge during the RSV season. An infant's gestational age may have been miscoded, as could have diagnoses for conditions associated with high risk of acquiring RSV. Therefore, some infants may have been improperly excluded from the analysis. RSV hospitalizations were identified through the presence of *an ICD-9-CM* diagnosis code on an inpatient medical claim rather than through RSV laboratory tests; therefore, some level of misclassification of RSV hospitalizations may have occurred. Unfortunately, given the limitations inherent to claims databases, the definition of RSV hospitalization used could not be validated against other clinical measures, such as RSV laboratory tests; however, a previous analysis found high concordance between RSV diagnosis in the emergency room setting and positive RSV tests.^24^ Additionally, health-care providers may not always test infants suspected of having RSV, in part because the American Academy of Pediatrics does not recommend routine testing once the RSV season has started.^25^

Furthermore, this analysis did not require a length of continuous enrollment; therefore, infants may have had <1 year of follow-up. Any RSV hospitalizations that occurred after these infants disenrolled from their health plans would not have been captured in this analysis. No statistical comparisons or multivariable analyses were conducted to assess significance or control for other factors. Finally, the proportion of infants admitted to the ICU and the proportion of infants requiring MV may be underestimated because of coding inconsistencies or undercoding as a result of bundling of in-hospital services.

## Conclusion

These results clearly demonstrate that the risk, severity and cost of RSV hospitalizations differ substantially for preterm vs full-term infants, and aggregate statistics combining infants of all gestational ages obscure the reality of the differences between preterm and full-term infants. Guidelines and policy debates would benefit from evidence that reflects the unique vulnerability of preterm and other high-risk infants for whom the clinical and cost consequences of RSV infections are likely to be much more significant than those for full-term infants.

## Figures and Tables

**Table 1:** Proportion of infants with a hospitalization for RSV by gestational age among Medicaid-insured and commercially insured infants

*Gestational age*	*Infants with hospitalization for RSV by age at first hospitalization, % (*n)	
	*<1 year old*^a^	*<90 days old*^a^
*Medicaid*		
Total (*N*=1 683 188)	1.8 (29 967)	0.9 (15 169)
Preterm (*n*=181 598)	3.0 (5480)	1.4 (2470)
<29 weeks (*n*=15 900)	3.8 (610)	0.5 (80)
29−30 weeks (*n*=10 846)	3.5 (381)	1.3 (138)
31−32 weeks (*n*=19 735)	3.3 (642)	1.4 (273)
33−34 weeks (*n*=43 850)	3.0 (1326)	1.5 (650)
35−36 weeks (*n*=91 267)	2.8 (2521)	1.5 (1329)
Full term (*n*=1 501 590)	1.6 (24 487)	0.8 (12 699)
		
*Commercial*		
Total (*N*=1 663 832)	1.0 (16 310)	0.5 (8577)
Preterm (*n*=147 234)	1.6 (2425)	0.7 (1099)
<29 weeks (*n*=10 300)	1.7 (177)	0.1 (6)
29–30 weeks (*n*=8035)	1.5 (120)	0.5 (37)
31–32 weeks (*n*=15 421)	1.5 (235)	0.5 (75)
33–34 weeks (*n*=37 231)	1.6 (601)	0.7 (276)
35–36 weeks (*n*=76 247)	1.7 (1292)	0.9 (705)
Full-term (*n*=1 516 598)	0.9 (13 885)	0.5 (7478)

Abbreviation: RSV, respiratory syncytial virus.

a Age at the time of first hospitalization for RSV.
