# Supplementary material for: Respiratory syncytial virus hospitalization outcomes and costs of full-term and preterm infants
Source: J Perinatol. 2016 Aug 4;36(11):990–6. doi: 10.1038/jp.2016.113 (PMC5090170; doi:10.1038/jp.2016.113)
Supplement: Supplementary Tables [file jp2016113x1.docx]

**Supplemental Tables**

**Supplemental Table 1. Distribution of Cost for Hospitalizations for RSV, Length of Stay for Hospitalizations for RSV, and Cost of Hospitalization for RSV During Which the Infant Was Admitted to the ICU**

|  | **N** | **Mean** | **SD** | **Median** | **IQR** |
| --- | --- | --- | --- | --- | --- |
| ***First-Year Cost of Hospitalizations for RSV (2014 US $)*** | | | | | |
| **Medicaid: <1 Year Old** |  |  |  |  |  |
| <29 wGA | 610 | $39,354 | $161,359 | $9,170 | $3,881–$24,968 |
| 29-30 wGA | 381 | $16,891 | $30,386 | $6,043 | $2,792–$14,330 |
| 31-32 wGA | 642 | $17,766 | $48,035 | $5,730 | $2,796–$14,467 |
| 33-34 wGA | 1,326 | $15,839 | $38,623 | $5,537 | $2,740–$11,883 |
| 35-36 wGA | 2,521 | $11,127 | $27,331 | $4,730 | $2,532–$9,731 |
| Full-term | 24,487 | $8,324 | $39,112 | $4,197 | $2,369–$7,678 |
| **Medicaid: <90 Days Old** |  |  |  |  |  |
| <29 wGA | 80 | $44,896 | $99,864 | $14,692 | $5,804–$47,672 |
| 29-30 wGA | 138 | $22,819 | $34,667 | $9,685 | $3,981–$21,807 |
| 31-32 wGA | 273 | $27,561 | $64,165 | $9,808 | $4,135–$29,162 |
| 33-34 wGA | 650 | $22,484 | $49,695 | $7,366 | $3,421–$18,304 |
| 35-36 wGA | 1,329 | $15,020 | $36,010 | $5,398 | $2,811–$12,810 |
| Full-term | 12,699 | $9,567 | $35,218 | $4,503 | $2,510–$8,453 |
| **Commercial: <1 Year Old** |  |  |  |  |  |
| <29 wGA | 177 | $40,813 | $150,983 | $12,115 | $6,444–$27,831 |
| 29-30 wGA | 120 | $34,032 | $113,058 | $7,267 | $5,086–$18,328 |
| 31-32 wGA | 235 | $23,782 | $51,908 | $8,305 | $5,316–$19,405 |
| 33-34 wGA | 601 | $19,931 | $40,831 | $7,965 | $4,545–$15,879 |
| 35-36 wGA | 1,292 | $16,299 | $32,645 | $7,254 | $4,437–$14,634 |
| Full-term | 13,885 | $10,570 | $30,860 | $5,887 | $3,799–$9,934 |
| **Commercial: <90 Days Old** |  |  |  |  |  |
| <29 wGA | 6 | $44,201 | $84,388 | $8,808 | $4,014–$24,961 |
| 29-30 wGA | 37 | $36,773 | $61,908 | $9,610 | $5,570–$27,573 |
| 31-32 wGA | 75 | $44,254 | $78,508 | $16,539 | $5,682–$40,182 |
| 33-34 wGA | 276 | $28,772 | $51,219 | $9,963 | $5,116–$23,510 |
| 35-36 wGA | 705 | $21,546 | $41,230 | $9,010 | $4,906–$19,984 |
| Full-term | 7,478 | $11,562 | $20,994 | $6,300 | $3,989–$11,011 |
| ***Length of Stay (days)*** | | | | | |
| **Medicaid: <1 Year Old** |  |  |  |  |  |
| <29 wGA | 610 | 9.2 | 13.5 | 5 | 3–10 |
| 29-30 wGA | 381 | 6.8 | 5.6 | 5 | 3–8 |
| 31-32 wGA | 642 | 6.1 | 6.9 | 4 | 3–7 |
| 33-34 wGA | 1,326 | 6.1 | 8.1 | 4 | 3–7 |
| 35-36 wGA | 2,521 | 5.4 | 4.9 | 4 | 3–6 |
| Full-term | 24,487 | 4.3 | 3.6 | 4 | 3–5 |
| **Medicaid: <90 Days Old** |  |  |  |  |  |
| <29 wGA | 80 | 11.4 | 11.7 | 8 | 5–13 |
| 29-30 wGA | 138 | 8.6 | 6.4 | 7 | 4–11 |
| 31-32 wGA | 273 | 8.0 | 9.3 | 6 | 4–10 |
| 33-34 wGA | 650 | 7.7 | 10.6 | 5 | 3–9 |
| 35-36 wGA | 1,329 | 6.4 | 6.1 | 5 | 3–8 |
| Full-term | 12,699 | 4.7 | 3.6 | 4 | 3–5 |
| **Commercial: <1 Year Old** |  |  |  |  |  |
| <29 wGA | 177 | 7.7 | 9.1 | 6 | 4–8 |
| 29-30 wGA | 120 | 6.0 | 6.9 | 4 | 3–6 |
| 31-32 wGA | 235 | 5.7 | 5.9 | 4 | 3–6 |
| 33-34 wGA | 601 | 5.3 | 4.9 | 4 | 3–6 |
| 35-36 wGA | 1,292 | 5.1 | 4.2 | 4 | 3–6 |
| Full-term | 13,885 | 4.1 | 3.2 | 3 | 3–5 |
| **Commercial: <90 Days Old** |  |  |  |  |  |
| <29 wGA | 6 | 6.5 | 6.9 | 4.5 | 2–6 |
| 29-30 wGA | 37 | 8.1 | 6.5 | 5 | 4–10 |
| 31-32 wGA | 75 | 7.6 | 7.6 | 5 | 3–9 |
| 33-34 wGA | 276 | 6.5 | 4.7 | 5 | 3–8 |
| 35-36 wGA | 705 | 6.0 | 4.9 | 4 | 3–7 |
| Full-term | 7,478 | 4.4 | 3.2 | 4 | 3–5 |
| ***RSV ICU Hospitalization Costs (2014 US $)*** | | | | | |
| **Medicaid: <1 Year Old** |  |  |  |  |  |
| <29 wGA | 190 | $90,168 | $275,141 | $30,339 | $11,501–$63,276 |
| 29-30 wGA | 108 | $35,482 | $42,541 | $17,757 | $7,703–$46,587 |
| 31-32 wGA | 134 | $48,563 | $90,086 | $24,815 | $9,808–$53,360 |
| 33-34 wGA | 281 | $43,886 | $68,429 | $19,767 | $8,414–$47,099 |
| 35-36 wGA | 364 | $37,697 | $62,724 | $18,412 | $6,624–$44,306 |
| Full-term | 1,954 | $35,623 | $133,320 | $12,797 | $4,997–$30,813 |
| **Commercial: <1 Year Old** |  |  |  |  |  |
| <29 wGA | 40 | $53,193 | $65,749 | $28,453 | $15,796–$51,955 |
| 29-30 wGA | 25 | $90,710 | $154,650 | $36,911 | $13,652–$74,518 |
| 31-32 wGA | 33 | $60,688 | $96,518 | $30,085 | $16,539–$53,101 |
| 33-34 wGA | 100 | $59,486 | $72,619 | $30,443 | $14,526–$84,650 |
| 35-36 wGA | 198 | $46,035 | $61,620 | $23,453 | $12,327–$57,746 |
| Full-term | 1,179 | $35,864 | $79,869 | $16,014 | $8,623–$37,317 |

SD, standard deviation; IQR, interquartile range

**Supplemental Table 2. Costs of Hospitalizations for RSV During Which the Infant Was Admitted to the ICU and Had Mechanical Ventilation**

|  | **N** | **Mean** | **SD** | **Median** | **IQR** |
| --- | --- | --- | --- | --- | --- |
| **Medicaid: <1 Year Old** |  |  |  |  |  |
| <29 wGA | 79 | $139,935 | $403,058 | $44,088 | $24,811–$93,523 |
| 29-30 wGA | 32 | $48,585 | $40,435 | $41,841 | $15,128–$65,205 |
| 31-32 wGA | 57 | $76,829 | $120,918 | $48,256 | $20,783–$83,906 |
| 33-34 wGA | 89 | $71,556 | $81,220 | $41,572 | $22,367–$90,485 |
| 35-36 wGA | 109 | $56,695 | $53,259 | $39,032 | $20,160–$78,859 |
| Full-term | 381 | $69,381 | $144,109 | $33,941 | $19,073–$70,731 |
| **Commercial: <1 Year Old** |  |  |  |  |  |
| <29 wGA | 11 | $118,245 | $91,129 | $92,866 | $46,754–$188,889 |
| 29-30 wGA | 9 | $179,563 | $225,775 | $138,280 | $68,516–$178,535 |
| 31-32 wGA | 6 | $102,621 | $69,219 | $81,438 | $56,810–$123,368 |
| 33-34 wGA | 29 | $84,898 | $71,269 | $76,341 | $39,965–$98,468 |
| 35-36 wGA | 39 | $90,492 | $69,848 | $78,136 | $45,727–$117,242 |
| Full-term | 151 | $89,464 | $151,715 | $61,702 | $32,701–$93,269 |

SD, standard deviation; IQR, interquartile range
